# Cancer-associated fibroblasts promote non-small cell lung cancer cell invasion by upregulation of glucose-regulated protein 78 (GRP78) expression in an integrated bionic microfluidic device

**DOI:** 10.18632/oncotarget.8232

**Published:** 2016-03-21

**Authors:** Ting Yu, Zhe Guo, Hui Fan, Jing Song, Yuanbin Liu, Zhancheng Gao, Qi Wang

**Affiliations:** ^1^ Department of Respiratory Medicine, The Second Hospital, Dalian Medical University, Dalian, China; ^2^ Department of Oncology, The Second Hospital, Dalian Medical University, Dalian, China; ^3^ Key Laboratory of Laboratory Medicine, Ministry of Education, Zhejiang Provincial Key Laboratory of Medical Genetics, Wenzhou Medical University, Wenzhou, China; ^4^ Department of Respiratory & Critical Care Medicine, the People's Hospital of Peking University, Beijing, China

**Keywords:** cancer-associated fibroblasts, lung cancer, invasion, GRP78, microfluidic chip

## Abstract

The tumor microenvironment is comprised of cancer cells and various stromal cells and their respective cellular components. Cancer-associated fibroblasts (CAFs), a major part of the stromal cells, are a key determinant in tumor progression, while glucose-regulated protein (GRP)78 is overexpressed in many human cancers and is involved in tumor invasion and metastasis. This study developed a microfluidic-based three dimension (3D) co-culture device to mimic an in vitro tumor microenvironment in order to investigate tumor cell invasion in real-time. This bionic chip provided significant information regarding the role of GRP78, which may be stimulated by CAFs, to promote non-small cell lung cancer cell invasion in vitro. The data showed that CAF induced migration of NSCLC A549 and SPCA-1 cells in this three-dimensional invasion microdevice, which is confirmed by using the traditional Transwell system. Furthermore, CAF induced GRP78 expression in A549 and SPCA-1 cells to facilitate NSCLC cell migration and invasion, whereas knockdown of GRP78 expression blocked A549 and SPCA-1 cell migration and invasion capacity. In conclusion, these data indicated that CAFs might promote NSCLC cell invasion by up-regulation of GRP78 expression and this bionic chip microdevice is a robust platform to assess the interaction of cancer and stromal cells in tumor environment study.

## INTRODUCTION

Lung cancer is one of the leading causes of cancerincidence and mortality in the world, accounting for more than 1.6 million new cases and 1.3 million deaths annually [1]. Histologically, non-small cell lung cancer (NSCLC) represents approximately 80% of all lung cancer cases. NSCLC is usually diagnosed at an advanced stage of disease and often metastases are present [2]. Thus, elucidation of the molecular mechanisms involved in NSCLC invasion and metastasis could lead to the emergence of novel diagnostic and therapeutic approaches. To date, accumulated evidence indicates that tumor lesions are composed of tumor parenchyma and stroma, two discrete but interactive cellular materials for cross talk and promotion of tumor growth [3, 4]. Indeed, tumor stroma plays a significant role in cancer evolution [5] by promoting tumorigenesis [6], cancer progression [7], invasion, [8] and chemoresistance [9] through a variety of mechanisms. As a major component in tumor stroma and microenvironment, cancer-associated fibroblasts (CAFs) are thought to be activated by tumor cells. CAFs are characterized by upregulated expression of a-smooth muscle actin (a-SMA), Vimentin, and fibroblast activation protein (FAP) [10-12]. Activation of CAF from regular fibroblasts induces multiple functional changes in CAF thereby promoting cancer development, such as facilitating angiogenesis, epithelial–mesenchymal transition (EMT) [13], dysfunction of the local immune system [14], and tumor cell proliferation, invasion, and metastasis [10–12]. However, the underlying molecular mechanisms of CAFs in promotion of tumor cell invasion and metastasis are poorly understood.

For example, the tumor microenvironment can mediate tumor cell growth by triggering stress responses through accumulating levels of the unfolded and/or misfolded proteins in the endoplasmic reticulum (ER) lumen, subsequently resulting in the unfolded protein response (UPR) [15]. The glucose-regulated protein GRP78, a stress-induced endoplasmic reticulum (ER) chaperone, is able to regulate the ER stress signaling pathways to induce the UPR by facilitating the folding and assembly of proteins, targeting misfolded proteins for ER associated degradation (ERAD), and regulating calcium homeostasis; thus, GRP78 serves as an ER stress sensor. GRP78 protein is usually expressed at the basal level in normal adult organs, such as the brain, lung and liver, but is significantly upregulated in various human cancers [16]. Moreover, overexpressed GRP78 in cancer cells is associated with tumor progression, a reduction in apoptosis, resistance to chemotherapy, and poor prognosis of several cancers [17–20]. Recently, studies have demonstrated that GRP78 expression was associated with invasion and metastasis of different types of cancer cells, such as gastric, prostate, and breast cancers [21–23].

To study tumor cell interactions with stromal cells in vitro, current cell models are limited. For example, traditional in vitro studies of three-dimensional (3D) tumor cell invasion were performed using a commercially available Transwell chamber by measuring the number of cells migrating vertically through a gel into a filter [24]. This system lacks real-time observation and it is inherently difficulty to assess tumor cell interaction with stromal cells directly. Thus, there is an urgent need to develop a reliable and efficient in vitro culture model to closely mimic the in vivo microenvironment of cancer metastasis. To this end, microfluidics bring a novel opportunity to spatially and temporally control tumor cell growth and stimuli and microfabricated devices have been used to facilitate the research need concerning the biology of cells [25–29]. The successful reconstitution of the lung tissue architecture on a microfluidic device indicates that biomimetic microsystems may potentially serve as a replacement for animal experiments [28]. Thus, in this study, we developed a microfluidic-based 3D co-culture device to recreate an in vitro tumor microenvironment to investigate the invasion capacity of cancer cells with respect to tumor cell interactions with stromal cells in real-time. This bionic chip could provide insightful information regarding the role of GRP78, stimulated by CAFs, in promotion of lung cancer cell invasion capacity.

## RESULTS

### Construction of the bionic invasion microfluidic device

**Figure 1:**
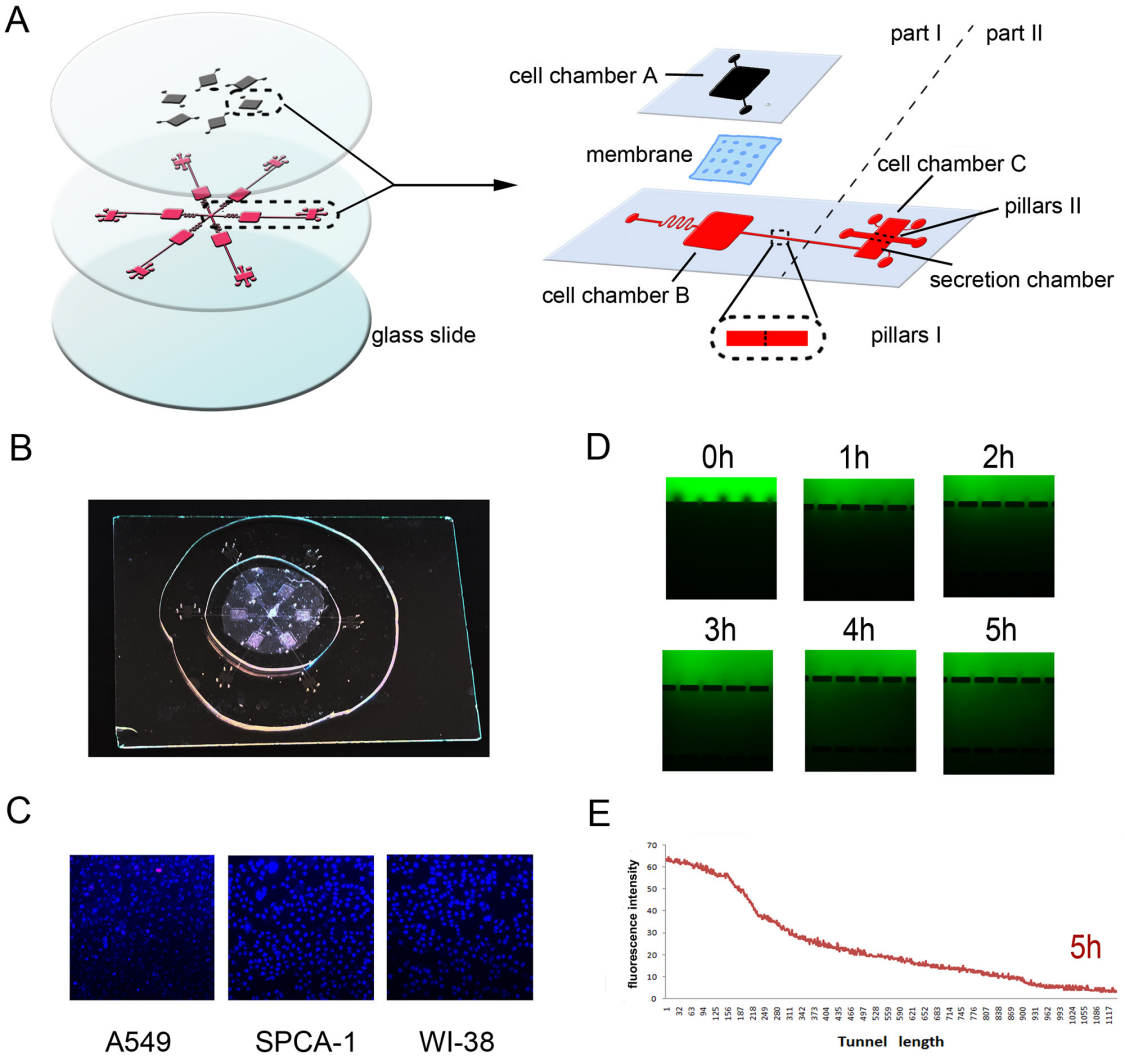
Design and illustration of the integrated bionic microfluidic chip **A.** A schematic diagram of the integrated microfluidic device (6 cm × 6 cm) to facilitate a non-contact cell co-culture model. **B.** A photograph of the integrated microfluidic device. **C.** Representative images of cells grown in the microfluidic device after 72h culture and Hoechst/PI staining. **D.** Representative images of immunofluorescence of FITC-dextran forming a concentration gradient across the basement membrane extract at different time points. **E.** Quantitative data on immunofluorescence concentration gradients at 5 hours in the device.

Microfluidic device was constructed to contain six chip units, which is used to assess cancer and stromal cell interactions and tumor cell invasion in vitro to mimic the in vivo conditions (Figure 1A). In this study, we applied this microfluidic device to assess lung cancer (A549 or SPCA-1) and fibroblast WI38 cell interaction by culturing them in the cell chambers A and B, respectively, for 72 h. Cell viability demonstrated that cells grew well within this device (Figure 1C). The migration channel filled with BME enabled formation of a stable concentration gradient. FITC was added into the growth medium to let it gradually diffuse into the basement membrane extract and spread to Channel C after 2 h, leading to a stable concentration gradient and maintaining over 4 h in the basement membrane extract. Figure 1DE measured such data in 5 h and showed data on tumor cell invasion over 48 h. As such, we replaced the CAFs’ medium in Channel C to maintain the concentration gradient of the basement membrane extract to be able to monitor cell invasion capacity.

### Transformation of fibroblast to CAFs using the chip

**Figure 2:**
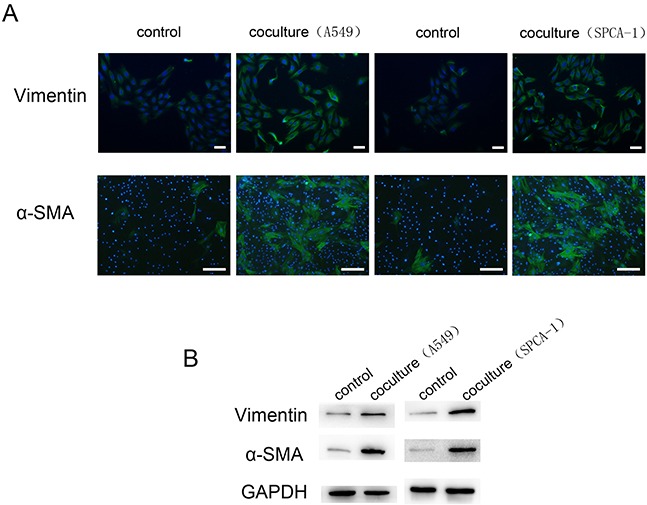
Activation of WI38 cells to CAFs after co-cultured with NSCLC cells **A.** Immunofluorescence detection of α-SMA and Vimentin expression in WI38 cells after co-cultured with A549 and SPCA-1 cells in a microfluidic chip for 72 h compared to that of control WI38 cells. Scale bar, 200 μm. **B.** Western blot. Level of α-SMA and Vimentin expression in WI38 cells after co-cultured with A549 and SPCA-1 in a Transwell system for 72 h compared to that of control WI38 cells.

In order to obtain CAFs for our in vitro study, we activated normal fibroblasts to CAFs by seeding A549 and SPCA-1 cells into the cell chambers in the chip unit part I and human lung fibroblasts WI38 into Chamber B (Figure 1A) and culturing them for 72 h. The data of the immunofluorescence assay revealed that WI38 cells co-cultured with NSCLC cells showed positive α-SMA and Vimentin expression compared to WI38 alone culture (Figure 2A). Moreover, we confirmed expression of these two markers in the Transwell WI38 co-cultured system using Western blot (Figure 2B).

### CAF-induced A549 and SPCA-1 cell migration and invasion through a three-dimensional invasion microdevice

**Figure 3:**
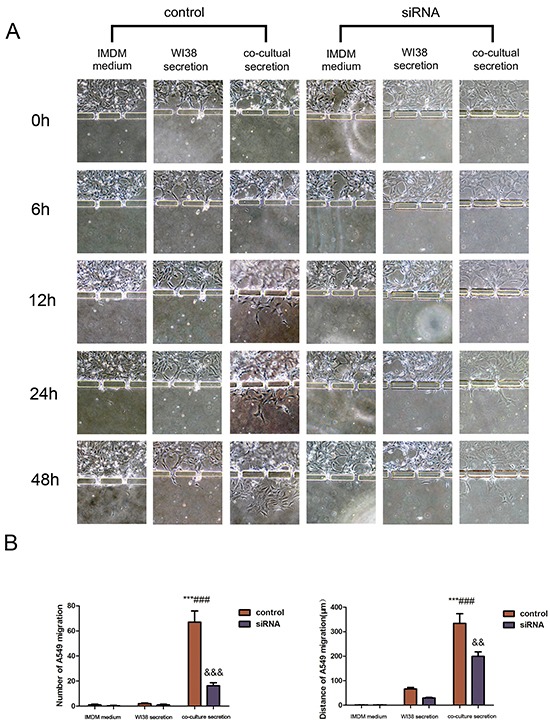
Changes in A549 cell invasion capacity in this 3D microfluidic device **A.** Representative images of the effect of control IMDM and WI38-conditioned and co-culture-conditioned growth media on A549 cell migration at different time points. **B.** Quantitative data showed the distance of cell migration and the number of migrating and invading cells under the different treatment conditions at 48h. Data are plotted as the mean ± SD of three separate experiments. ***P < 0.001 versus IMDM control; ###P < 0.001 versus WI38 secretion control, &&P < 0.01 and &&&P < 0.001 versus co-culture medium control.

**Figure 4:**
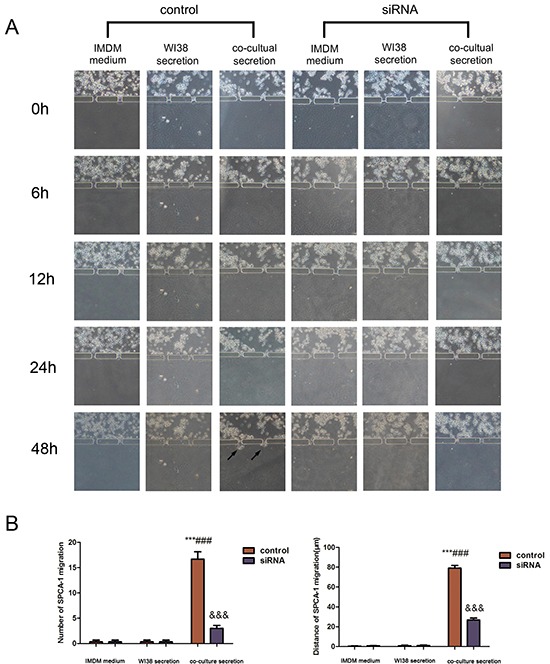
Changes in SPCA-1cell invasion capacity in the 3D microfluidic device **A.** Representative images of the effect of control IMDM and WI38-conditioned and co-culture-conditioned growth media on SPCA-1cell migration at different time points. Arrows indicated invading tumor cells. **B.** Quantitative data showed the distance of cell invasion and the number of invading cells under the different treatment conditions at 48h. Data are plotted as mean ± SD of three separate experiments. ***P < 0.001 versus IMDM medium control; ###P < 0.001 versus WI38 secretion control, and &&&P < 0.001 versus co-culture medium control.

This microdevice contains two units, Part I and II. Part I also contains two chambers to separate A549 and SPCA-1 cells from WI38 cells in chamber B. After WI38 transformed into CAF in co-culture in part I, we cultured NSCLC (A549 and SPCA-1) cells as control group and GRP78 knockdown NSCLC cells as siRNA experimental group in Chamber C. After cells adhered to the chamber, secretion from above flowed into the secretion chamber. Multiple inducers (IMDM, WI38 secretion, or co-culture medium) were added into secretion chamber and tumor cell migration was recorded using an inverted phase contrast microscope over a period of 48 h. We found that when treated with co-culture secretions, tumor cells migrated towards the microchannels where the inducers’ concentration was highest and digested BME and then invaded towards the secretion chamber. Tumor cell migration and invasion behavior first appeared at 6 h after addition with co-culture secretions, but was not observed in tumor cells treated with IMDM medium and WI38 secretion. The migration and invasion capacity of NSCLC cells was lower after knocking down of GRP78 expression (Figure 3A and Figure 4A). Quantitative data showed that tumor cells induced by co-culture secretions migrated faster, whereas tumor cells with knocking down of GRP78 expression showed less number and migration or invasion distance than that of control cells (Figure 3B and Figure 4A).

### CAF induction of GRP78 expression in A549 and SPCA-1 cells

**Figure 5:**
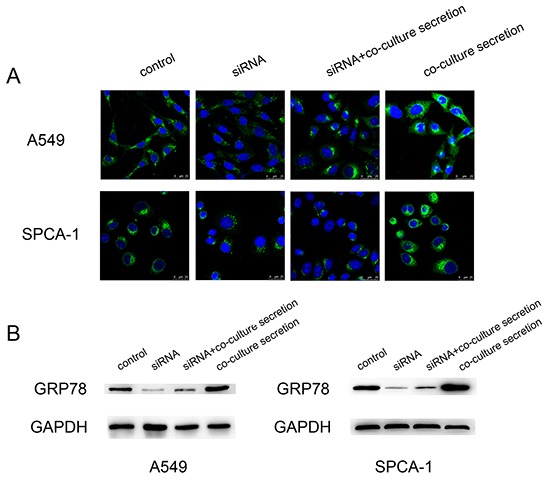
Upregulation of GRP78 expression in NSCLC cell lines after cultured with the CAFs-conditioned medium **A.** Representative immunofluorescence images of GRP78 expression in A549 and SPCA-1 cells at different treatment groups. Scale bar, 25 μm. **B.** Western blot. The data showed expression levels of GRP78 protein in A549 and SPCA-1 cells at different groups.

To explore the underlying mechanism, we examined whether CAF regulates expression of GRP78 protein. After knocking down GRP78 expression in tumor cells using siRNA, Western blot and immunofluorescence data revealed that this siRNA was effective (Figure 5AB). However, when we cultured these cells with CAF secretion for an additional 24 h, Western blot and immunofluorescence data showed that CAF secretion induced a pronounced increase in GRP78 expression in these tumor cells compared with the corresponding controls (both negative control and siRNA-only control)(Figure 5AB). These data indicate that CAF was able to induce GRP78 expression in A549 and SPCA-1 cells.

### Traditional Transwell assay to confirm findings from microdevice data

**Figure 6:**
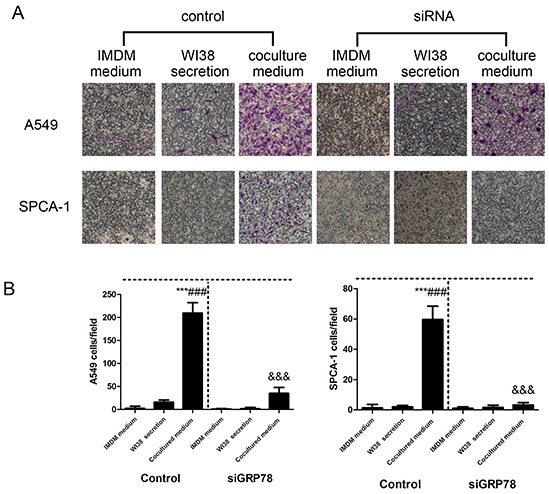
Detection of tumor cell invasion capacity using the traditional Transwell assay **A.** Representative images of the effect of control IMDM and WI38-conditioned and co-culture-conditioned growth media on control group and GRP78 knocked down group of NSCLC cells. **B.** Quantitative data showed the number of invading A549 and SPCA-1cells under different treatment conditions. Data are plotted as mean ± SD of three separate experiments. ***P < 0.001 versus IMDM medium control; ###P < 0.001 versus WI38 secretion control, and &&&P < 0.001 versus co-culture medium control.

In order to verify the microdevice is reliable, we repeated the tumor cell invasion assay using a traditional Transwell system. As shown in Figure 6A, Transwell assay revealed that NSCLC cells induced by co-culture conditional medium had a significant increase in invasion capacity compared to the control medium, whereas the number and invasion of NSCLC cells after down-regulated GRP78 expression were shown to be less than those of controls (Figure 6AB).

## DISCUSSION

The tumor microenvironment favors tumor immune privilege as well as inducing proliferation. Resistance to apoptosis is found in cancer cells and various stromal cells, such as fibroblasts, vascular cells and inflammatory cells [33]. A previous study demonstrated that CAFs, activated by cancer cells, can secrete ECM components, a variety of growth factors, and chemokines to promote tumor cell growth, invasion, and metastasis, besides their direct interaction with cancer cells [34]. To date, the molecular mechanisms by which CAFs promotes tumor invasion and metastasis remains to be defined. Compared to normal tissue, many human cancer cells showed upregulation of GRP78 protein expression. GPR78 is also implicated in oncogenesis, cancer progression, and drug resistance [35]. To further explore the mechanisms of NSCLC progression, our current study developed a bionic chip to allow the co-culture of human lung fibroblasts WI38 cells with human lung adenocarcinoma cells to mimic NSCLC cell migration and invasion in vivo. We first activated normal human fibroblasts to CAFs using this microdevice and showed increased level of the myofibroblast markers a-SMA and Vimentin, consistent with other previous studies [12]. These findings indicated that our bionic chip was an excellent platform to investigate the cell–cell interactions that mimic the in vivo environment.

We then assessed CAFs-conditioned growth medium in the promotion of NSCLC cell invasion and demonstrated that NSCLC cells exhibited increased migration. Our findings suggest that CAFs-secreted stuff was able to more strongly influence the motility of NSCLC cells than that of normal fibroblasts. Furthermore, we found that GRP78 expression was also upregulated in NSCLC cells after culture with CAFs-conditioned growth medium. The number, migration and invasion distance of invading NSCLC cells after GRP78 knockdown was significantly lower than those of control cells. We also found that both number and distance of SPCA-1 cell invasion were less than those of A549 cells, indicating that the migratory capacity of varying tumor cells induced by CAF was different. Following these findings using this novel microfluidic device to test tumor cell invasion capacity, we also confirmed our microdevice data using the traditional Transwell system. We demonstrated that our microdevice has several advantages over the traditional Transwell assay; for example, our chip is able monitor tumor cell migration across the BME in real-time. Moreover, our chip can greatly reduce cell numbers and amount of reagents needed. Our microdevice can also process variously treated cells under multiple inducers simultaneously, which can greatly reduce experimental error. Our current microdevice data are consistent with the previous studies showing that GRP78 protein is a regulator of tumor invasion in many kinds of human cancers [36,37]. In this sense, we propose that CAFs play a key role in progression of human lung adenocarcinoma cells via increase in GRP78 expression.

However, our current study does have some limitations. The interaction of CAFs with NSCLC cells in this bionic chip culture system and future research should incorporate additional stroma cell types into this system to study the cell–cell interactions taking place. Moreover, the underlying molecular mechanism by which CAFs interact with NSCLC cells still requires further study. Utilization of this novel microfluidic chip has some limitations, like all other techniques, so we may not use it to replace other technologies, but just add one. In summary, we developed an integrated co-culture bionic chip to assess tumor cell invasion in real time. Our current data showed that conditioned growth medium obtained from co-culture of cancer-associated fibroblasts and NSCLC cells promoted NSCLC cell invasion mediated by the up-regulation of GRP78 expression. These findings suggest that GRP78 may be a novel target in future treatment of NSCLC.

## MATERIALS AND METHODS

### Design and fabrication of the bionic invasion chip

The schematic illustration of the integrated microenvironment chip is shown in Figure 1A and the manufacturing process was described in our previous study [30]. Specifically, this microchip was fabricated with poly-dimethylsiloxane (PDMS) (Dow, Corning, MI, USA) using standard soft lithography methods with replica molding of PDMS against the masters. The upper and lower layers of this microchip and a glass slide were irreversibly bonded together in a sequence via oxygen plasma surface treatment (150 mTorr, 50 W, 20 s) [31, 32].

This device contains six units of chip and each unit is composed of two parts, one for cellular co-culture (part I) and another one for cell invasion (part II). Part I consisted of two layers of PDMS material, between which there is a 5-μm pore polycarbonate membrane (Nuclepore, Whatman, Buckinghamshire, UK) segmenting into two-cell culture chambers (namely, A and B). Non-small cell lung cancer A549 and SPCA-1 cell lines were seeded into Chamber A at approximately 1 × 10^3^ cells/cm^2^, while 2 × 10^3^ cells/cm^2^ fibroblast WI38 cells into Chamber B, respectively, to reach a ratio of fibroblasts to NSCLC cells of 2:1. It is structured as a non-contacting co-culture system to simulate tumor and stromal cell interactions to mimic the tumor microenvironment in vivo. The gap of pillar A is 8μm in size which is smaller than the size of WI38 cells, which can effectively block WI38 cells from crossing the cell chamber, whereas macromolecules secreted from WI38 cells are easily able to cross the chambers. Part II is also composed of two chambers (secretion chamber and cell chamber C) and a migration channel. The migration channel has a dimension of 40μm x 500 μm x 2mm (H x W x L), and several micropillars with 20μm gaps embedded into both edges. This design can only allow the cultrex basement membrane extract (BME; R&D Systems, Minneapolis, MN, USA) to flow into the migration channel but not flow into the adjacent chambers. For example, non-small cell lung cancer A549 and SPCA-1 cell lines were seeded into Chamber C and, following cell adherence to the base, secretion from the upstream allows flow into the secretion chamber constantly, which could induce tumor cells to digest BME and invade into the secretion chamber. This chip provides a scaffolding structure within which cancer cells and fibroblasts interact directly in three-dimensions (3D) mode [26] but without a direct contact each other. The cell-basement membrane extract (BME) mixture was used to seed into each cell culture chamber to separate these types of cells. In order to form a stable concentration gradient of CAFs’ secretion in this chip to induce tumor cells invasion, this chip has been designed to connect cell culture chambers with an input and a syringe pump linking to each chamber in order to control the flow of a culture medium from the upper chamber to the downstream chamber to form a concentration gradient of the conditional medium or tracer (such as a fluorescence dye). A finished integrated bionic microfluidic device is shown in Figure 1B. Cells cultured in part I were stained with Hoechst/propidium iodide (PI; Figure 1C). We followed the diffusion process with FITC-conjugated goat anti-mouse IgG at a dilution of 1:200 (Jackson ImmunoResearch, West Grove, PA, USA) and reviewed under a fluorescence microscope.

### Cell lines and culture

Human lung adenocarcinoma A549 and SPCA-1 cell lines were obtained from American Type Culture Collection (Manassas, VA, USA) and cultured in RPMI-1640 medium (Gibco, Long Islands, NY), while human lung fibroblast WI38 cells were also obtained from ATCC and cultured in IMDM (Gibco) at 37°C in a humidified atmosphere of 5% CO_2_. These cell culture media were also supplemented with 10% fetal bovine serum (FBS, Hyclone, Logan, UT, USA) and penicillin (100 U mL^−1^), and streptomycin (100 μg mL^−1^).

### Immunofluorescence

We performed immunofluorescence to detect expression of specific biomarkers α-SMA and Vimentin of CAFs, and GRP78 expression in A549 and SPCA-1 cells cultured in this chip and Transwell, respectively. In brief, cells were rinsed in phosphate buffered saline (PBS)for three times and fixed in 4% paraformaldehyde for 15 min and then permeabilized in 0.1% Triton X-100 (AppliChem, Switzerland) for 20 min. After three washes, cells were blocked in 5% bovine serum albumin (BSA, Sigma, St Louis, MO, USA) solution in PBS for 1h at 37°C. To confirm fibroblasts transformed into CAFs, WI38 cells were incubated with an anti-α-SMA antibody (Abcam, UK) and anti-Vimentin antibody (Abcam), respectively for 12 h at 4°C.

To detect level of GRP78 expression after induced by CAFs, A549 and SPCA-1 cells were incubated with ananti-GRP78 antibody (Abcam) and subsequently with an Alexa Fluor® 488-conjugated secondary antibody (donkey anti-mouse IgG, Invitrogen, Carlsbad, CA, USA) at 37°C for 1h. Cells were then counterstained with DAPI (Sigma) and reviewed under a fluorescent microscope with a confocal imaging system (Confocal Laser Scanning Microscope CLSM, Leica TCS SP5 II, Germany).

### Production of CAF-conditioned growth medium using the transwell assay

To assess tumor-stromal cell interactions in vitro, we utilized an indirect contact co-culture system of a Transwell apparatus with a 0.4-μm-pore membrane (six-well plate; Corning, Corning, NY, USA). Tumor cells were added into the upper chamber of the Transwell insert and WI38 cells were added into the lower chamber. After incubation for three days, the co-cultured medium was collected and centrifuged to remove cellular debris, and the supernatants were frozen at −80°C and used as a chemoattractant for tumor cell invasion assay. Co-cultured WI38 cells were collected for Western blot analysis of α-SMA and Vimentin proteins.

### Protein extraction and western blot

Cells were harvested, lysed in an RIPA buffer with fresh addition of 10 mg/ml phenylmethanesulphonyl fluoride and 1% (v/v) cocktail protease inhibitor (Sigma) for 30 min. The protein concentration was determined by the BCA assay. Protein lysates were then separated by sodium dodecyl sulfate-polyacrylamide gel electrophoresis (SDS-PAGE) and transferred onto nitrocellulose membranes (Millipore, Billerica, MA, USA). For Western blotting, the membranes were blocked in 5% fat-free dry milk solution in PBS and then incubated with primary antibodies against grp78 (1:1000;Abcam), α-SMA (1:200;Abcam), Vimentin (1:1000;Abcam), GAPDH(1:5000; Proteintech Group Inc., Chicago, IL, USA) and further with a secondary antibody in the Super Signal West Pico Kit (Thermo Fisher Scientific Inc., USA). The levels of interested proteins were analyzed by Gel-Pro4.0 software (Media Cybernetics, Rockville, MD,USA).

### RNAi interference

The siRNA used to knockdown Grp78 expression was designed and synthesized by Invitrogen (Shanghai, China) and DNA sequences of siRNA duplex were 5′-CCAAAGACGCUGGAACUAUTT-3′ and 5′-AUAGU UCCAGCGUCUUUGGTT-3′. Transfection was conducted by using Lipofectamine^™^ 3000(Invitrogen) according to the manufacturer's instructions. Briefly, cells were plated in 6-well plates and cultured for 24 h and then transfection complex containing 5 μg siRNA was added into cell culture and cells were further cultured for 72 h at 37°C.The level of GRP78 expression was determined by Western blot.

### Tumor cell transwell invasion assay

Matrigel(Corning) was used to pre-coat the filters with 8-μm pore size between the upper and bottom chambers of the Transwell apparatus (Corning). After the Matrigel solidified at 37°C, cancer cells were seeded into the upper chambers and then co-cultured medium, WI38 cell secretion, and IMDM were added into the bottom Transwell chamber. Cells were incubated at 37°C overnight. Cells remaining in the surface of the filter were swabbed with a cotton swab and cells invaded into the surface of the bottom filter were fixed with 100% methanol for 10 min and stained in Giemza stain (Sigma Chemical Co) for 10min and then washed with distilled water. The number of cells invaded into the lower surface of the polycarbonate filter was counted at 100× magnification under a light microscope. Each type of cells was assayed in triplicate and repeated at least twice. Invading cancer cells were then collected for immunofluorescence and Western blotting.

### Statistical analysis

Data were expressed as the means ± standard deviation and the difference among groups was analyzed by analysis of variance using SPSS13.0 for Windows software (SPSS, Chicago, IL, USA). A *p* value of ≤ 0.05 was considered statistically significant.
